# Sequence and Characteristics of Atomic Ordering in Ni_2_Mn_1−x_Cu_x_Ga Ferromagnetic Shape Memory Alloys

**DOI:** 10.3390/ma15238529

**Published:** 2022-11-30

**Authors:** Concepció Seguí

**Affiliations:** Departament de Física, Campus Ctra, Universitat de les Illes Balears, Valldemossa, km 7,5, E-07122 Palma, Spain; concepcio.segui@uib.es

**Keywords:** ferromagnetic shape memory alloys, Ni_2_MnGa, atomic ordering, saturation magnetization

## Abstract

Post-quench atomic reordering processes undergone by Ni_2_Mn_1−x_Cu_x_Ga alloys have been characterized in detail. The obtained results corroborate the hypothesis that proposes an atomic ordering process additional to the B2↔L2_1_ one, consisting of the relocation in the Mn sublattice of Cu atoms misplaced by quench in the Ni sublattice. In addition, the results suggest that the ordering of the Cu atoms and the L2_1_ ordering can occur in different sequences depending on the starting state of order. The analysis of the saturation magnetization validates the occurrence of two types of atomic movements; the values corresponding to different post-quench stages have been compared with those calculated for different atomic configurations, supporting the relocation mechanism of Cu atoms as the most plausible mechanism. The effect of the quenching temperature on the reordering processes has been also studied, and an assessment of the degree of quenched disorder is provided, suggesting the existence of an order–disorder transition associated with Cu atoms ordering. Finally, the effect of the Cu amount has been analyzed, confirming that a greater amount of Cu intensifies the process associated to ordering of Cu atoms, which takes place even in martensite.

## 1. Introduction

Ferromagnetic shape memory alloys (FSMA) have drawn much attention in recent years, since they add to the unique capabilities of conventional shape memory alloys and other attractive properties resulting from the coupling between the magnetic and structural phase transitions, such as magnetic-field-induced strain, magnetic shape memory, or magnetocaloric effect (MCE) [1,2,3]. The most studied FSMAs are Heusler-type, particularly near-stoichiometric Ni_2_MnGa alloys, that undergo thermoelastic martensitic transformation (MT) from the cubic L2_1_ parent phase (austenite) to a less symmetric phase (martensite). Much work has been devoted to the study of the structural and magnetic properties of Ni-Mn-Ga-based alloy systems, often focusing on their composition dependence. In that sense, the addition of a fourth element strongly affects both the MT (T_MT_) and Curie temperatures (T_C_) and allows for tuning the relative position of the magnetic and structural transformations. This is of utmost importance, since several functional properties, such as MCE, require the existence of a magnetization jump between phases. Among doping elements, Cu has been extensively studied, since the sequence of magnetic/structural transformations can be tailored by changing the Cu content and the element it replaces. In summary, Cu substituting Mn or Ga increases the T_MT_ and decreases both the austenite and martensite T_C_ [4,5]; instead, Cu substituting Ni lowers T_MT_ and raises T_C_ [6].

**Figure 1:**
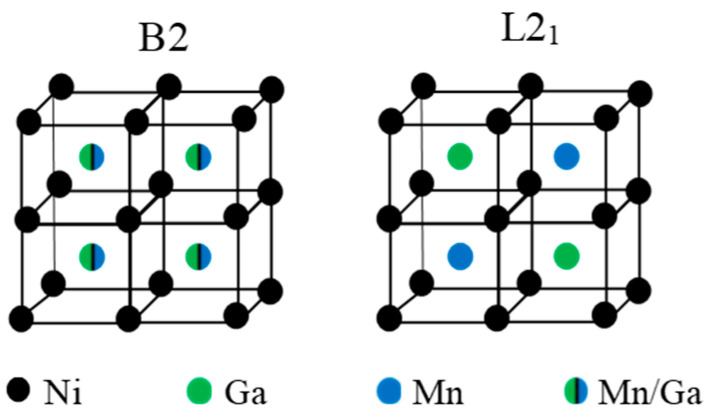
Atomic occupancies of the cubic lattice for B2 and L2_1_ order in ternary Ni_2_MnGa alloy.

Ni-Mn-Ga-based alloys show a marked sensitivity to the degree of atomic order [7,8,9,10,11,12,13,14,15]. Ni-Mn-based alloys solidify from the melt to the cubic B2 structure, and the ordered L2_1_ structure was formed through a B2↔L2_1_ ordering reaction (around 1100 K for ternary Ni_2_MnGa [13]). A schematic representation of the atomic occupancy in the cubic lattice for the different order states B2 and L2_1_ for ternary Ni_2_MnGa alloy is shown in Figure 1. As a general rule, high-temperature quenching followed by post-quench aging has proven to be effective to control the order degree; neutron diffraction measurements have confirmed [9,16] that quenching from temperatures around the ordering transition produces a loosely ordered L2_1_ structure, so that a certain degree of atomic disorder is retained. L2_1_ order can be progressively improved up to the equilibrium value by post-quench ageing at temperatures at which atomic diffusion is possible. It is a widespread result that T_C_ increases with L2_1_ order as a consequence of the variation of the magnetic moment of the alloys, depending on the position of the Mn atoms, which couple antiferromagnetically when they are nearest-neighbors (Mn atoms on the Ga positions) and ferromagnetically when they are next-nearest-neighbors (Mn atoms in the Mn sublattice) [17]. The MT temperatures are also modified by changes in atomic order, although there is no unique trend: T_MT_ has been observed to increase (as in ternary Ni-Mn-Ga [8,16]) or to drop (as in Co− or In-doped Ni-Mn-Ga [9,11,12]) with increasing atomic order. Instead, the Ni-Mn-Sn system shows almost no changes after quenching from high temperature [10]. It is worth mentioning that the post-quench reordering process is often evidenced by the appearance of a broad exothermic calorimetric peak which appears at temperatures well above the MT, only in the first heating run after quench [7,8].

In a recent work, the influence of atomic order on the martensitic and magnetic transformations undergone by Ni_2_Mn_1−x_Cu_x_Ga FSMAs was investigated [18]. Both the MT and Curie temperatures decrease by quench and raise during post-quench aging, but these temperatures do not evolve in parallel: T_MT_ increases from the start of aging while T_C_ begins to rise at a later stage in the aging process. Furthermore, during the first post-quench heating, Ni_2_Mn_1−x_Cu_x_Ga alloys display two consecutive DSC exothermic peaks, indicative of two thermal processes, each of which can be related to the evolution of a transition temperature: the one appearing at lower temperatures can be associated to a process which produces an increase in the MT temperatures, while the one occurring at higher temperatures accounts for another process that results in an increase of Curie temperature. This phenomenon, never reported before, was analyzed and discussed in detail in [18,19], basing on its most significant characteristics: first, ternary Ni-Mn-Ga, Co-doped Ni-Mn-Ga, and Ni_2−x_Cu_x_MnGa alloys display a single exothermic peak, while all tested Ni_2_Mn_1−x_Cu_x_Ga alloys present the two post-quench exothermic peaks; the role of Cu substituting Mn is therefore decisive to the phenomenon [18]. Second, the peak common to all alloys disappears after quenching from temperatures well below the B2↔L21 transition, the lowest temperature exothermic peak being the only one remaining after quench from temperatures where the L21 order is complete. Finally, the detailed study of the kinetics of the post-quench aging processes [19] reveals that both can be described by a first order reaction model with similar activation energies around 1.1 eV, with the vacancies retained by high temperature quenching playing a crucial role in these processes.

According to the above, and having ruled out the formation of structures other than the Heusler X_2_YZ [18], the occurrence of the process undergone by all alloys that is responsible for the change in Curie temperature is attributed to the improvement of L2_1_ order, due to exchange of Mn and Ga atoms located at antisites after quench; for the other process, which only occurs in Ni_2_Mn_1−x_Cu_x_Ga alloys and underlies the rise of martensitic transformation temperatures, diffusion of Cu atoms, misplaced in the Ni sublattice after quench, towards their most favorable sites in the Mn sublattice is proposed as the responsible mechanism [18]. Unfortunately, X-ray or TEM–electron diffraction does not allow us to distinguish what type of atom occupies the available sites, withneutron diffraction remaining as the only option to discern site occupation. 

While a detailed study is carried out by means of neutron diffraction to corroborate the atomic movements proposed as the most probable hypothesis, there are still several unknowns, whose clarification will undoubtedly help to better understand the mechanisms of atomic ordering in Ni_2_Mn_1−x_Cu_x_Ga alloys.

In this work, the characterization of the post-quench atomic reordering processes that Ni_2_Mn_1−x_Cu_x_Ga alloys undergo has been deepened. All the obtained results are consistent with the hypothesis formulated that proposes, for these alloys, an atomic ordering process additional to the B2↔L2_1_ one, consisting of the relocation in the Mn sublattice of Cu atoms misplaced by quench in the Ni sublattice. In addition, the results suggest that the ordering of the Cu atoms and the L2_1_ ordering can occur in different sequences depending on the starting state of order.

## 2. Materials and Methods

Polycrystalline Ni_2_Mn_1−x_Cu_x_Ga (x = 0.12–0.44) alloy ingots were prepared by induction melting in argon atmosphere, using the appropriate quantities of the constituent elements Ni, Mn, and Cu of 99.99 % purity and Ga of 99.9999 % purity (Neyco Vacuum and Materials, Vanves, France). The alloys are labelled according to the Cu atomic percent as Cu% (% = 25·x). The ingots were melted at least twice to improve homogeneity and subjected to homogenization heat treatment consisting of 24 h at 1170 K in a vacuum quartz tube, followed by quenching in water at room temperature. The composition of the homogenized alloys was checked by energy-dispersive x-ray spectroscopy (EDS, Bruker X-flash detector 4010, Bruker GmbH, Berlin, Germany) in a scanning electron microscope (Hitachi S-3400 N, Hitachi High-Tech Ltd., Tokyo, Japan); in all cases, the content of the different elements turned out to differ by less than 5% from the nominal compositions.

The samples used in the different experimental techniques were cut using a diamond saw from the already-homogenized ingots. Long range atomic order of the samples was modified by means of additional thermal treatments, consisting of annealing for 1 h at temperatures T_WQ_ = 570–1170 K, followed by quench into water at room temperature, and subsequent post-quench ageing, carried out by continuous heating up to a maximum of 700 K. 

The martensitic and magnetic transitions, as well as the reordering processes, have been monitored by Differential Scanning Calorimetry measurements (DSC 2920, TA Instruments, New Castle, DE, USA), at a heating rate of 5 K/min, using prismatic samples with mass around 100 mg. From the DSC curves, the transition temperatures and the exchanged heats are determined, with the latter integrating the heat flow curves after the proper baseline correction. To minimize the error made when calculating the heat released during the exothermic reordering peaks, the curve corresponding to the second heating was taken as baseline and subtracted from the curve obtained after quenching.

Magnetization measurements under magnetic fields up to 7 T have been performed in a vibrating sample magnetometer (VSM; magnetic platform from Cryogenic Ltd. CFMS, London, UK), using bulk prismatic samples with mass around 10 mg. 

## 3. Results and Discussion

### 3.1. Ordering Sequences

**Figure 2:**
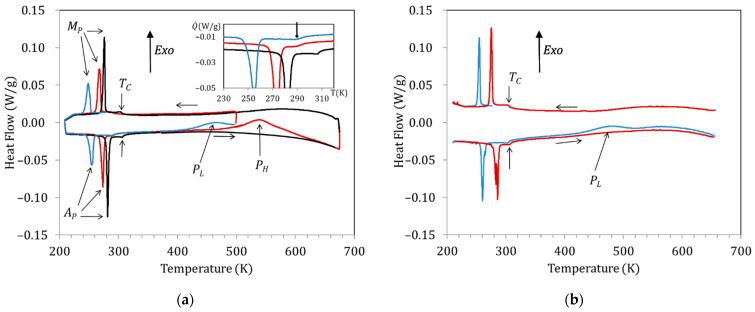
DSC curves obtained for a Cu5 sample (**a**) water quenched from 1020 K: just-quenched (blue curve), after overcoming P_L_ (red curve), and after passing P_H_ (black curve). (**b**) Just after quench from 770 K (blue curve) and (**b**) quenched from 770 K: just-quenched (blue curve) and after heating up to 670 K (red curve).

Figure 2a shows the DSC curves obtained for a Cu5 sample water quenched from 1020 K. The exothermic and endothermic peaks observed at low temperatures correspond to the forward (on cooling; peak temperature M_P_) and reverse (on heating; peak temperature A_P_) MT, respectively, whereas the anomaly around 300 K is associated with the magnetic transition taking place in the austenitic phase ($T_{C}^{\;}$); during post-quench heating, two broad exothermic peaks are observed, labelled as P_L_ and P_H_. Figure 2a displays the curves obtained during cooling down to 210 K and subsequent heating up to 500 K of the just-quenched sample (blue curve), cooling after overcoming P_L_ and heating up to 670 K (red curve), and cooling after passing P_H_ followed by heating up to 670 K (black line). Comparison of the blue and red curves with the black one in the heating range 400 K–670 K shows that the exothermic peaks P_L_ and P_H_ are irreversible, as they do not appear in subsequent heating runs. Both the martensitic and magnetic transition temperatures are found to drop by quench and to increase during post-quench aging, but while there is a significant change in M_P_ and A_P_ after heating above P_L_ (compare blue and red curves), $T_{C}^{\;}$ does not change until the sample is heated to above P_L_, as shown in the inset (red and black curves). In order to discern the effects of the processes underlying P_L_ and P_H_, we will name the different aging states as quenched (WQS; before blue curve), intermediate-aged (IAS; before red curve), and full-aged (FAS; before black curve).

On its turn, Figure 2b shows the DSC curves obtained for the same Cu5 alloy after quench from 770 K. As explained in the introduction, after quench from temperatures well below the B2↔L21 transition, the P_H_ peak is supressed and only P_L_ remains. Consistent with the changes in transition temperatures observed in Figure 2a, it can be seen in Figure 2b that quench from 770 K affects the MT temperatures but not the Curie temperature. 

**Figure 3:**
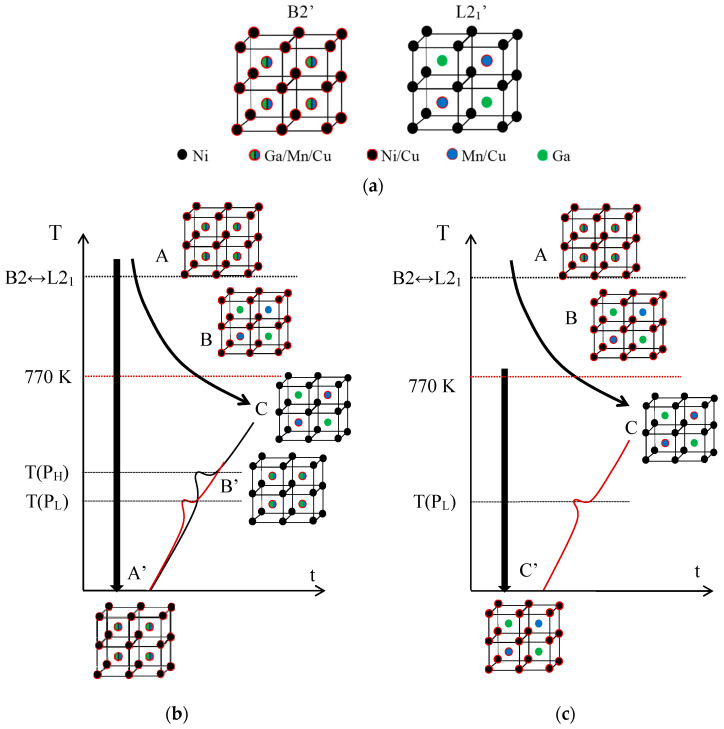
Crystal structure of the austenite with the proposed site occupation in each state of the sequences, as explained in the text. (**a**) Atomic occupancies for B2′ and L2_1′_ order in Ni_2_Mn_1−x_Cu_x_Ga alloys. Sequences of ordering after quench (**b**) from the B2′ temperature domain; and (**c**) from below the B2↔L2_1_ temperature.

Sticking to the hypothesis that the P_H_ peak is associated with the L2_1_ ordering (that is, with the switch of Mn and Ga atoms from the improper to their proper sublattices), while P_L_ accounts for the emplacement in the Mn sublattice of Cu atoms, misplaced in the Ni sublattice after quench, it results from Figure 2 that these two ordering processes take place in a different sequence depending on the starting state. This is summarized in Figure 3, which illustrates the crystal structure of the austenite with the proposed site occupation in each state and the different ordering sequences.

First, Figure 3a shows the atomic occupancies in the states of order B2′ and L2_1_‘ for the present Ni_2_Mn_1−x_Cu_x_Ga alloys. In the B2’ ordered state we expect to find Cu atoms in the Mn and Ga sublattices, but also in the Ni sublattice, according to the formulated hypothesis. Likewise, the most ordered structure L2_1_’ corresponds to the Cu atoms in the sites of the Mn that it replaces. In Figure 3b, states A and C correspond to B2′ and L2_1′_ respectively, and A’ is obtained by quenching from the temperature domain of B2’; although it certainly holds a substantial L2_1_‘ order, it retains a certain degree of B2’ order, which is shown in the figure. If from state A’ heating proceeds continuously, during the P_L_ process ordering of the Cu atoms located in Ni sites, towards the Mn sublattice, occurs, leading to a state called B’ in which the disorder between Mn and Ga atoms remains. On further heating, during the P_H_ process, the Mn-Ga ordering takes place, resulting in the ordered structure L2_1_’ (state C). Instead, below the ordering temperature B2↔L2_1_, the stable state is called B in Figure 3c, in which the Mn and Ga atoms occupy their own sublattices, but some Cu atoms remain misplaced in the Ni sites. Quenching from that state retains the disorder of the Cu atoms (C’ state), and subsequent heating leads, through the P_L_ process, to the ordered C state.

### 3.2. Magnetic Behavior

**Figure 4:**
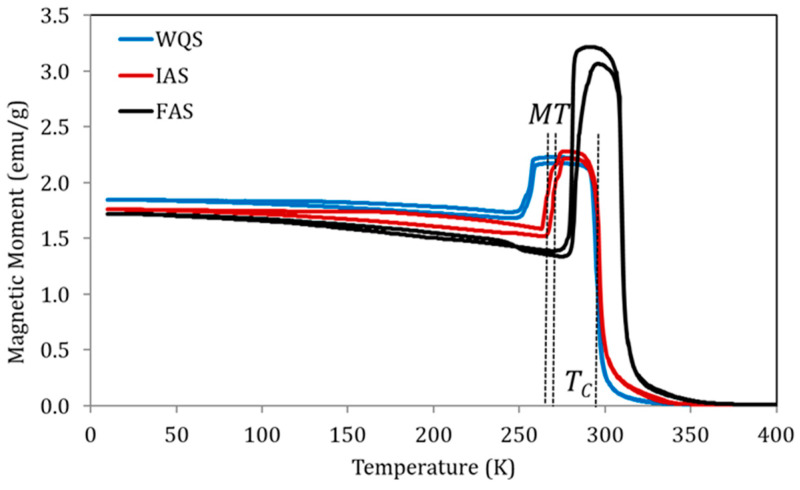
Magnetization vs temperature curves under a constant magnetic field of 5 mT for samples of alloy Cu5 quenched from 1020 K in three different post-quench ageing states: just quenched (WQS), intermediate-aged (IAS), and full-aged (FAS).

Figure 4 shows the magnetization measured at a constant magnetic field of 5 mT, as a function of temperature, for samples of alloy Cu5 quenched from 1020 K in three different post-quench ageing states as defined above: just quenched (WQS), intermediate-aged (IAS), and full-aged (FAS). In line with the previously established relationship between the processes underlying P_L_ and P_H_ and the variation of transition temperatures, the IAS sample shows MT temperatures higher than WQS but with no T_C_ changes, while between IAS and FAS samples, an increase in T_C_ was also observed.

**Figure 5:**
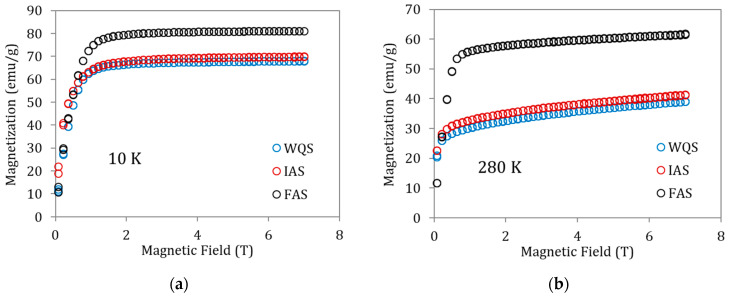
Magnetization vs. magnetic field curves for samples of alloy Cu5 quenched from 1020 K in WQS, IAS, and FAS ageing states recorded at (**a**) 10 K and (**b**) 280 K.

To better understand the magnetization changes associated with these states of order, magnetization vs. magnetic field curves for the above specimens were also recorded at 10 K (ferromagnetic martensite, Figure 5a) and 280 K (ferromagnetic austenite, Figure 5b). The saturation magnetization increases from WQS to FAS—i.e., with atomic order degree—but it is worth paying attention to the fact that the increase in magnetization is very small between WQS and IAS (overcoming P_L_), with the true increase being produced when passing from IAS to FAS (that is, after overcoming P_H_). With the behavior being the same in Figure 5a,b, the M(μ_o_H) values for ferromagnetic austenite at 280 K are clearly lower than those observed for ferromagnetic martensite at 10 K. This can easily be attributed to the fact that 280 K is not far enough below the Curie temperature to have complete magnetic order; in fact, the gap (T_C_ −280 K) is different for WQS, IAS, and FAS samples. Trying to approach more realistic values of the saturation magnetization in austenite, the Kuz’min [20] formula can be applied

(1) m(T)=M(T)M(0)=[1−s(1−δ)32−(1−s)(1−δ)52]13

(2) M(0)=M(T)[1−s(1−δ)32−(1−s)(1−δ)52]13

where δ=(TC −T)TC  is the reduced temperature difference, M(T) is the spontaneous magnetization at temperature T, M(0) is the saturation magnetization at 0 K, and s is a parameter related to the shape of the magnetization versus temperature curves; in [12,21], s = 0.1 was successfully used for austenite. The Curie temperatures for WQS, IAS, and FAS were taken as 300 K, 301 K, and 316 K, as depicted in Figure 4. The saturation magnetization of ferromagnetic martensite and austenite was determined from the Arrott plots, based on the mean field theory applied to magnetism which predicts that M2 vs. (HM) should be linear below the Curie temperature. High field data have been fitted to a linear function that is extrapolated to HM=0 to obtain the saturation magnetization. The obtained values, together with the calculated austenite 0 K values as indicated above, are given in Table 1, where it can be seen that the magnetic moment experiences a slight rise after overcoming the process associated with P_L_ and a significantly greater rise when overcoming the process P_H_.

The saturation magnetization per formula unit that would correspond at 0 K to the full-ordered Ni_2_Mn_1−x_Cu_x_Ga can be computed from

(3) $$\mu_{\mathrm{sat}}\left(\frac{\mu_{B}}{\mathrm{fu}}\right)=2\cdot \mu_{\mathrm{Ni}}^{\mathrm{Ni}}+\left(1-x\right)\mu_{\mathrm{Mn}}^{\mathrm{Mn}}+x\cdot \mu_{\mathrm{Cu}}^{\mathrm{Mn}}+\mu_{\mathrm{Ga}}^{\mathrm{Ga}}$$

The values for $\mu_{X}^{Y}$ (magnetic moment of X atoms in the Y sublattice) can be found in [22] to be $\mu_{\mathrm{Ni}}^{\mathrm{Ni}}=0.32{\;\mu }_{B}$, $\mu_{\mathrm{Mn}}^{\mathrm{Mn}}=3.37{\;\mu }_{B},\;\mu_{\mathrm{Cu}}^{\mathrm{Mn}}=-0.03{\;\mu }_{B}$ and $\mu_{\mathrm{Ga}}^{\mathrm{Ga}}=-0.05{\;\mu }_{B}$. Particularizing in the Cu5 alloy (x = 0.2) to which the previous results refer,

(4) $$\mu_{\mathrm{sat}}\left(\frac{\mu_{B}}{\mathrm{fu}}\right)=2\cdot \mu_{\mathrm{Ni}}^{\mathrm{Ni}}+0.8\cdot \mu_{\mathrm{Mn}}^{\mathrm{Mn}}+0.2\cdot \mu_{\mathrm{Cu}}^{\mathrm{Mn}}+\mu_{\mathrm{Ga}}^{\mathrm{Ga}}=3.28$$

a value somewhat higher than those listed in Table 1 for sample FAS, meaning that not even FAS corresponds to an optimal degree of atomic order. To explain the measured magnetic moments for states WQS, IAS, and FAS, we will refer to the atomic occupation of the sublattices in case it is different from the perfectly ordered one. Clearly, the greatest changes in magnetization are associated with the position of the Mn atoms, due to antiferromagnetic coupling of the Mn-Mn pairs when Mn atoms are not located at the proper sites in the Heusler structure [22]. Since $\mu_{\mathrm{Mn}}^{\mathrm{Ga}}=-3.43{\;\mu }_{B}$ and $\mu_{\mathrm{Ga}}^{\mathrm{Mn}}=-0.01{\;\mu }_{B}$ [22], a fraction α of Mn-Ga atoms exchange would lead to

(5) $$\mu_{\mathrm{sat}}\left(\frac{\mu_{B}}{\mathrm{fu}}\right)=2\cdot \mu_{\mathrm{Ni}}^{\mathrm{Ni}}+\left(0.8-\alpha \right)\cdot \mu_{\mathrm{Mn}}^{\mathrm{Mn}}+\alpha \cdot \mu_{\mathrm{Ga}}^{\mathrm{Mn}}+0.2\cdot \mu_{\mathrm{Cu}}^{\mathrm{Mn}}+\left(1-\alpha \right)\cdot \mu_{\mathrm{Ga}}^{\mathrm{Ga}}+\alpha \cdot \mu_{\mathrm{Mn}}^{\mathrm{Ga}}=\left(3.2-6.76\alpha \right)$$

accounting for a decrease of $6.76\alpha \;\mu_{B}$. In this sense, the lower the L2_1_ order degree, the more Mn-Mn pairs are coupled antiferromagnetically, and therefore, the resulting saturation magnetization is lower. In addition to the Mn-Ga (L2_1_) disorder, the placement of the Cu atoms will be considered to explain the measured values of $\mu_{\mathrm{sat}}$. Following [22], for the Ni_2_Mn_0.8_Cu_0.2_Ga alloys, the configurations listed below can be considered, ranked from the most (*i*) to the least favorable energetically (*v*):

*i.*  Ni_2_ (Mn_0.8_Cu_0.2_) Ga
*ii.*  Ni_2_ (Mn_0.8_Ga_0.2_) (Ga_0.8_Cu_0.2_)
*iii.*  (Ni_1.8_Cu_0.2_) (Mn_0.8_Ni_0.2_) Ga
*iv.*  (Ni_1.8_Cu_0.2_) (Mn_0.8_Ga_0.2_) (Ga_0.8_Ni_0.2_)
*v.*  (Ni_1.8_Ga_0.2_) (Mn_0.8_Ni_0.2_) (Ga_0.8_Cu_0.2_)

The *v* configuration is discarded as being highly unfavorable, and for the remaining ones, we can evaluate the saturation magnetic moment using $\mu_{\mathrm{Ni}}^{\mathrm{Mn}}=0.15{\;\mu }_{B},{\;\mu }_{\mathrm{Ni}}^{\mathrm{Ga}}=0.12{\;\mu }_{B};{\;\mu }_{\mathrm{Cu}}^{\mathrm{Ni}}=0.04{\;\mu }_{B},\;\mu_{\mathrm{Cu}}^{\mathrm{Ga}}=-0.01{\;\mu }_{B}$ and $\mu_{\mathrm{Ga}}^{\mathrm{Ni}}=-0.03{\;\mu }_{B},{\;\mu }_{\mathrm{Ga}}^{\mathrm{Mn}}=-0.01{\;\mu }_{B}$ [22]. Configuration *ii*, that is, Cu atoms misplaced in the Ga sublattice, yield an increase of the saturation magnetization, thus it does not seem to be a likely configuration after quench (WQS); instead, *iii* and *iv* produce comparable decrease of of $\mu_{\mathrm{sat}}$ with respect to *i*. In this way, the slight increase in $\mu_{\mathrm{sat}}$ experienced between WQS and IAS states is compatible with the relocation of Cu atoms, misplaced by quench in the Ni sublattice, towards the Mn sublattice, in agreement with the hypotheses which was formulated in [18]. Among *iii* and *iv*, the configuration *iii* is more stable according to the first principles calculations in [22], so the following atom allocation is proposed to explain the measured $\mu_{\mathrm{sat}}$ values:

WQS: (Ni_2−β_Cu_β_) (Ni_β_Cu_0.2−β_Mn_0.8−α_Ga_α_) (Ga_1−α_Mn_α_)

(6) $$\begin{array}{ll} \mu_{\mathrm{sat}}\left(\frac{\mu_{B}}{\mathrm{fu}}\right) & =\left(2-\beta \right)\cdot \mu_{\mathrm{Ni}}^{\mathrm{Ni}}+\beta \cdot \mu_{\mathrm{Cu}}^{\mathrm{Ni}}+\beta \cdot \mu_{\mathrm{Ni}}^{\mathrm{Mn}}+\left(0.2-\beta \right)\cdot \mu_{\mathrm{Cu}}^{\mathrm{Mn}}+\left(0.8-\alpha \right)\cdot \mu_{\mathrm{Mn}}^{\mathrm{Mn}}+\alpha \cdot \mu_{\mathrm{Ga}}^{\mathrm{Mn}}+\left(1-\alpha \right)\cdot \mu_{\mathrm{Ga}}^{\mathrm{Ga}}+\alpha \cdot \mu_{\mathrm{Mn}}^{\mathrm{Ga}}\\ & =3.28-6.76\cdot \alpha -0.1\cdot \beta \end{array}$$

IAS: Ni_2_ (Cu_0.2_Mn_0.8−α_Ga_α_) (Ga_1−α_Mn_α_)

(7) $$\mu_{\mathrm{sat}}\left(\frac{\mu_{B}}{\mathrm{fu}}\right)=2\cdot \mu_{\mathrm{Ni}}^{\mathrm{Ni}}+0.2\cdot \mu_{\mathrm{Cu}}^{\mathrm{Mn}}+\left(0.8-\alpha \right)\cdot \mu_{\mathrm{Mn}}^{\mathrm{Mn}}+\alpha \cdot \mu_{\mathrm{Ga}}^{\mathrm{Mn}}+\left(1-\alpha \right)\cdot \mu_{\mathrm{Ga}}^{\mathrm{Ga}}+\alpha \cdot \mu_{\mathrm{Mn}}^{\mathrm{Ga}}=3.28-6.76\cdot \alpha$$

FAS: Ni_2_ (Cu_0.2_Mn_0.8_) Ga

(8) $$\mu_{\mathrm{sat}}\left(\frac{\mu_{B}}{\mathrm{fu}}\right)=2\cdot \mu_{\mathrm{Ni}}^{\mathrm{Ni}}+0.8\cdot \mu_{\mathrm{Mn}}^{\mathrm{Mn}}+0.2\cdot \mu_{\mathrm{Cu}}^{\mathrm{Mn}}+\mu_{\mathrm{Ga}}^{\mathrm{Ga}}=3.28$$

Evidently, from a quantitative point of view, it is a mere approximation, since it is obvious that the Mn-Ga exchanges take place throughout the entire aging process, although their rate be maximum in the temperature domain where P_H_ occurs; in the same way, other atomic movements cannot be ruled out, which in any case would be less relevant. For this reason, it would be very daring trying to deduce, from the values in Table 1, the fractions α and β of misplaced atoms. At most, from the comparison between the values for WQS, IAS, and FAS, we can estimate α = 0.07–0.08 after quench, and remaining α = 0.01–0.02 in FAS.

### 3.3. Evaluation of Order Degree for Different T_WQ_

**Figure 6:**
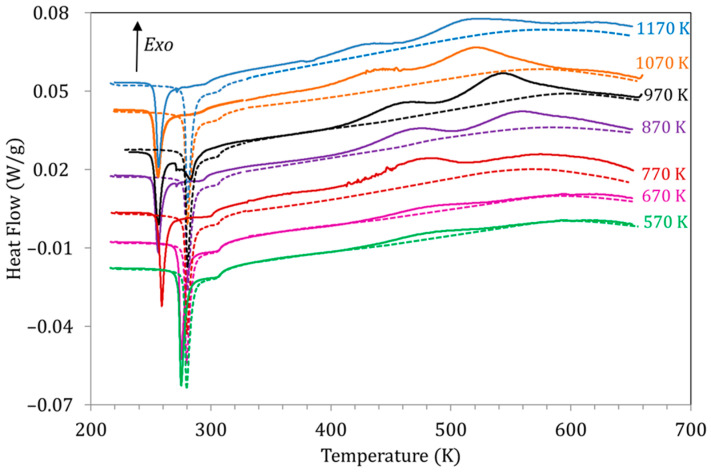
DSC curves obtained for alloy Cu5 during the first (solid lines) and second (dashed lines) heating runs after quench from different T_WQ_.

A relevant characteristic of the studied processes is their behavior for different quenching temperatures (T_WQ_). In this sense, the most outstanding observation, as already mentioned, is the suppression of P_H_ for T_WQ_ below 770 K. However, T_WQ_ has other interesting effects, both on the structural and magnetic transitions as well as on the exothermic processes P_L_ and P_H_. Figure 6 shows the DSC curves obtained for alloy Cu5 during the first (solid lines) and second (dashed lines) heating runs after quench from different T_WQ_. As we know, both the Curie and MT temperatures recorded in the first heating run (quenched state) fall relative to the second heating (ordered, stable state); it can be observed in Figure 6 that the value of the drops ∆T_C_, ∆M_P_ and ΔA_P_ depends on T_WQ_; all temperature drops are larger the higher T_WQ_, but ∆T_C_ becomes stable for T_WQ_ above 970 K (that is, coincident with the B2↔L2_1_ temperature) and is null for quenches from 670 K and below, while ∆M_P_ and ∆A_P_ stabilize for quenching above 870 K, and even for quenching temperatures below 570 K, non-zero ∆M_P_ and ΔA_P_ are observed (see also Figure 8 in [18]). The behavior of ∆T_C_, ∆M_P_, and ∆A_P_ supports that while the process responsible for the variation of Curie temperature is closely related to L2_1_ ordering, the changes in the MT temperatures are generated in a process that takes place during quench from temperatures well below the B2↔L2_1_ ordering reaction. 

**Figure 7:**
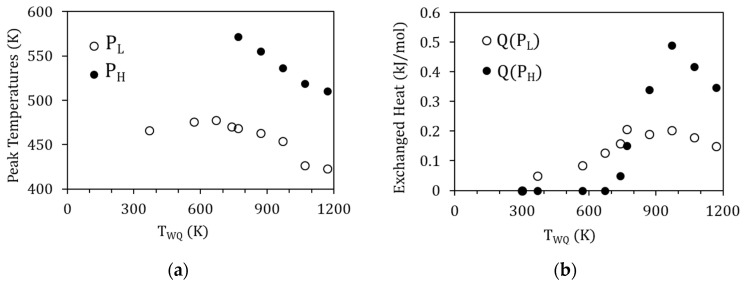
(**a**) Peak temperature of P_L_ and P_H_ as a function of the quenching temperature T_WQ;_ (**b**) evolution with T_WQ_ of the heat released during P_L_ and P_H_ (Q(P_L_) and Q(P_H_), respectively).

The evolution of the exothermic peaks P_L_ and P_H_ as a function of T_WQ_ can also be seen in Figure 6. Along with the suppression of P_H_ for T_WQ_ below 770 K, both peaks P_L_ and P_H_ are observed to shift towards higher temperatures as T_WQ_ is lowered, as depicted in Figure 7a. This would indicate that the related processes require less energy to start after quenching from higher temperatures, which, as discussed in [19], can be related to the crucial role of vacancies in assisting the reordering processes. On its turn, Figure 7b shows the evolution with T_WQ_ of the heat released during the processes underlying P_L_ and P_H_, computed from the area under the DSC curves as explained in Section 2. Q(P_L_) and Q(P_H_) increase as T_WQ_ increases, meaning that these processes involve more energy after quenching from higher temperatures.

To understand the meaning of the heat released in the exothermic peaks, let us take as an example the case of P_H_, related to the improvement of the L2_1_ order. The heat released would be proportional to the enhancement of the order degree achieved during the process giving rise to P_H_, and ultimately to the degree of quenched disorder, which is clearly dependent on T_WQ_.

**Figure 8:**
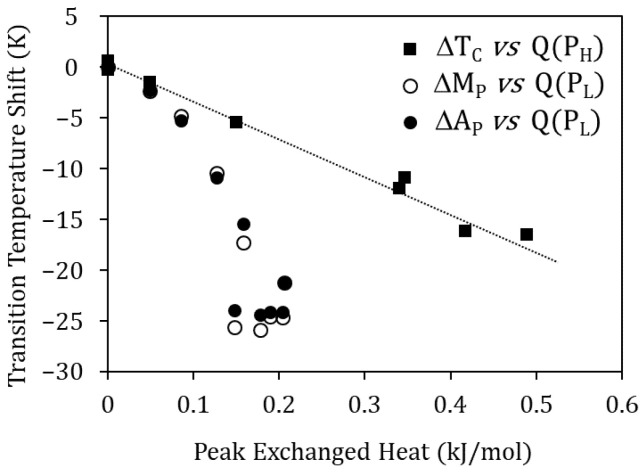
Curie temperature drop ∆T_C_ due to quench from T_WQ_ vs. heat released in the P_H_ peak, and MT temperatures drop (∆M_P_ and ∆A_P_) vs. heat released at peak P_L_.

Moreover, Figure 8 shows that the Curie temperature drop ∆T_C_ due to quench from T_WQ_ and the heat released in the P_H_ peak are proportional, suggesting their common origin: the higher T_WQ_, the lower the degree of atomic order after quench, giving rise to a greater drop in T_C_ and to a reordering process that involves more energy. The reasoning is the same for P_L_, as it can be seen in Figure 8 relating the heat released at peak P_L_, with the MT temperatures droping due to quench from T_WQ_. In this case, the invoked reordering process is the relocation of Cu atoms, misplaced in the Ni sublattice, towards their most favorable sites in the Mn sublattice.

In either case, the heat released in the exothermic processes P_L_ and P_H_ would account for the degree of quenched-disorder according to:

(9) $$s\left(T_{\mathrm{WQ}}\right)=1-\frac{Q\left(T_{\mathrm{WQ}}\right)}{Q_{\max}}$$

**Figure 9:**
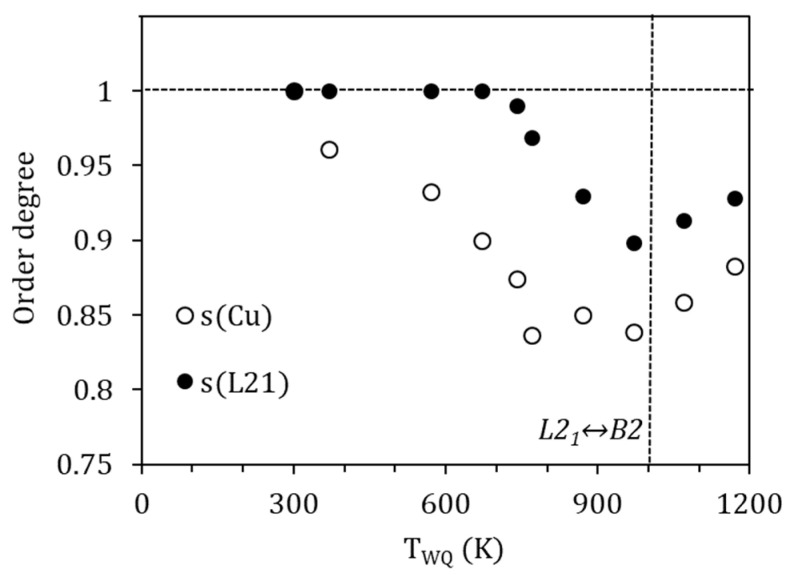
Calculated degrees of order after quenching from different temperatures.

$s\left(T_{\mathrm{WQ}}\right)$ being the degree of order after quench from T_WQ_, $Q\left(T_{\mathrm{WQ}}\right)$ the heat released in the corresponding exothermic reordering peak, and $Q_{\max}$ the maximum heat for the ordering process; that is, the energy required to go from complete disordered to full ordered states. For the ordering B2↔L2_1_, to which we relate P_H_, it is possible to find in the literature values obtained through ab-initio calculations; Ref. [23] provides a value of 50 meV/atom (4.82 kJ/mol) for the stoichiometric Ni_2_MnGa alloy. Instead, for the ordering of the Cu atoms in the corresponding sublattice, we do not have reference values, but we can get an approximation from [22], where the site preference of Cu-doped Ni_2_MnGa alloys is analyzed from the first principles. In this work, a relative free energy difference of 0.24 mRy/atom (3.26 meV/atom) between the configuration Ni_2_(Mn_0.95_Cu_0.05_) Ga and (Ni_1.95_Cu_0.05_)(Mn_0.95_Ni_0.05_) Ga is calculated, thus a difference of 13.06 meV/at (1.26 kJ/mol) can be inferred between the most stable Ni_2_ (Mn_0.8_Cu_0.2_) Ga configuration and the disordered (Ni_1.8_Cu_0.2_) (Mn_0.8_Ni_0.2_) Ga one. Using these values as Q_max_ in Equation (9) we can calculate the degree of order after quenching from different temperatures, with the results shown in Figure 9.

It is interesting to note that the values obtained for the degree of order L2_1_ (P_H_) after quench are similar to those evaluated in the literature (see, for example, [8,9,10]), with a minimum of s(L2_1_)~0.9 after quench from temperatures around the B2↔L2_1_ temperature. It can be also observed, and it is quite a common feature, that the degree of order improves for higher temperatures above the order–disorder one, which is attributable to the role of quenched-in vacancies in facilitating diffusion.

On its turn, the degree of order associated with the site occupancy of Cu atoms takes minimum values around 0.85 due to quench from temperatures above 770 K. This would imply that around 15% of the Cu atoms are displaced from their most favorable sites after quench. In addition, as it was already clear from the evolution of the temperature shifts, a perfect order is not achieved, even when quenching from temperatures as low as 370 K. 

Comparing the evolution of this degree of order and the L2_1_ one, an order–disorder transition would be expected to occur at temperatures around 770 K. However, unlike the B2↔L2_1_ transition, this eventual order–disorder transition is not accompanied by latent heat, nor has it been possible to detect its presence by other experimental techniques, so it must be attributed to a continuous transition.

### 3.4. Effect of Cu Content

**Figure 10:**
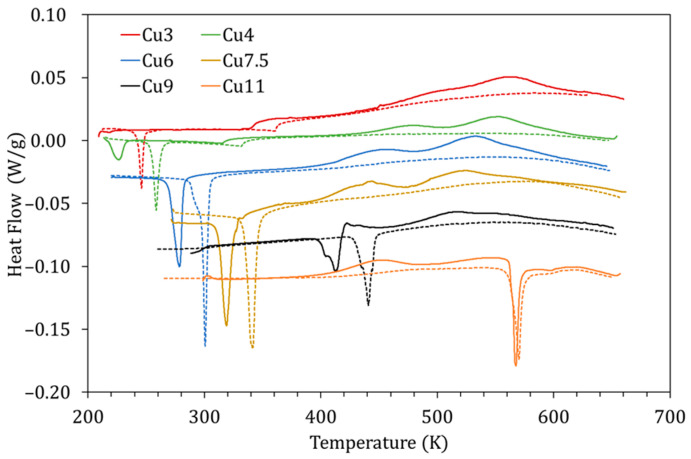
DSC curves obtained for alloys with different Cu content (3–11 at% Cu) quenched from 1020 K during the first (solid lines) and second (dashed lines) heating runs.

Given that the mechanism proposed for P_L_ is based on the diffusion of Cu atoms towards their most energetically favorable sites, it is natural to assume that the characteristics of this process will be enhanced in alloys with more Cu. Figure 10 shows the DSC curves obtained for alloys with different Cu content (3–11 at% Cu) quenched from 1020 K during the first (solid lines) and second (dashed lines) heating runs. In Figure 10, along with the variation of the MT and Curie temperatures owing to Cu content [5], the exothermic peaks P_L_ and P_H_ are also observed to change.

**Figure 11:**
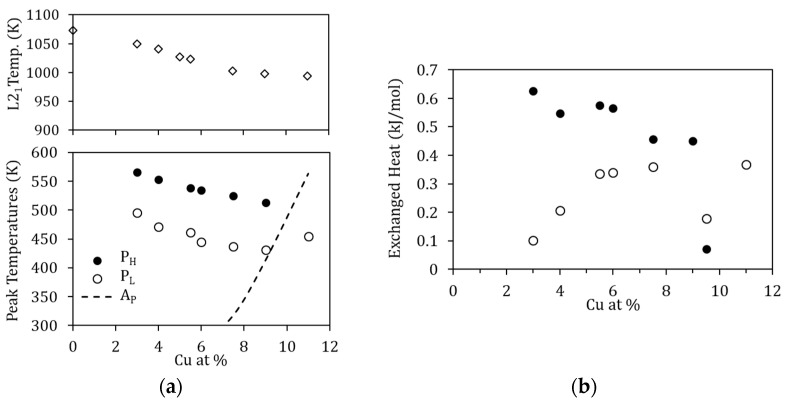
(**a**) Peak temperature of P_L_ and P_H_, together with the temperature of the B2↔L2_1_ ordering transition, as a function of the Cu-content; the dashed line shows the reverse MT temperature; (**b**) Heat released during the processes P_L_ and P_H_ as a function of the Cu-content.

The influence of the amount of Cu is evidenced through different magnitudes. First, the temperatures at which P_L_ and P_H_ occur decrease as the amount of Cu increases; this is depicted in Figure 11a, together with the temperature of the B2↔L2_1_ transition. The reason as to why this happens is not straightforward. On the one hand, since the processes are thermally activated, the peak temperature evolution could be related to composition-dependent changes in the kinetic parameters. On the other hand, since the B2↔L2_1_ temperatures also decrease with increasing Cu, quenching from the same temperature (1020 K) will lead to different degrees of quenched-disorder for the different compositions, modifying the characteristics of the reordering processes.

Second, in Figure 10, a phenomenon never reported to date in the literature can be observed; for the alloy with the highest Cu content (Cu11), displaying the highest MT temperatures of the set, one exothermic, transient, peak appears in martensite. This is an unprecedented observation, since it has always been assumed that diffusion-related post-quench atomic reordering only takes place in austenite (being usual to observe a related exothermic peak [7,8]). On the other hand, atomic diffusion in martensite, and the atomic rearrangement that it entails, have always been related to the phenomenon of martensite stabilization [24] (no thermal effect associated with having been reported to date). However, as can be seen in Figure 10, there is no stabilization of martensite, since the MT transformation temperatures are the same in the first and second heating runs. Incidentally, no stabilization of martensite was detected after keeping a sample for 3 h at 470 K, so this alloy, with high MT temperature, remains very stable at high temperatures. Although it could be doubted whether the detected exothermic peak is P_L_ or P_H_, the fact that it remains after quench from 770 K, well below its L2_1_ ordering temperature, suggests that it is P_L_, and so is quoted in Figure 11a. This is a very interesting experimental fact that should be studied in detail independently, but in the meantime, it gives rise to the idea that, unlike the L2_1_ ordering (Mn-Ga pairs), the reordering process involving the Cu atoms can take place in martensite.

Lastly, as might be expected, the heat released during the processes P_L_ and P_H_ changes with Cu content, increasing with Cu in the case of Q(P_L_) and increasing with Mn content for Q(P_H_). This is shown in Figure 11b and can be interpreted as an indication that increasing the Cu (Mn) content increases the probability of finding misplaced atoms after quenching, and therefore the overall energy required for relocation increases. Nevertheless, as already mentioned, for the different Cu-content, the degree of disorder retained by quench from 1020 K will be different, as will the reference energy values, which prevents estimating the fraction of atoms that are relocated in the corresponding processes.

Detection of the disorder caused by the exchange between Ni and Cu atoms requires special measurement setups like neutron diffraction. Thus, a detailed neutron powder diffraction study is on the way, with the goal of elucidating the atomic site occupancies during post-quench aging of the Ni_2_Mn_1−x_Cu_x_Ga alloys. It will also be interesting to explore whether other families of quaternary alloys present similar ordering sequences in order to establish if it is a more general phenomenon and, if so, what would be the characteristics that give rise to it.

## 4. Conclusions

In this work, the characterization of the post-quench atomic reordering processes undergone by Ni_2_Mn_1−x_Cu_x_Ga alloys has been deepened. All the obtained results are consistent with the formulated hypothesis that proposes for these alloys an atomic ordering process additional to the B2↔L2_1_ one, consisting of the relocation in the Mn sublattice of Cu atoms misplaced by quench in the Ni sublattice. In addition, the results suggest that the ordering of the Cu atoms and the L2_1_ ordering can occur in different sequences depending on the starting state of order.

Likewise, the analysis of the saturation magnetization validates that after quenching two types of atomic movements take place consecutively, of which only one involves diffusion of Mn. The saturation magnetization values obtained have been compared with those calculated for different atomic configurations, corroborating the relocation mechanism of Cu atoms that constitutes the working hypothesis.

Additionally, the effect of the quenching temperature on the reordering processes has been studied; an assessment of the degree of quenched disorder is provided, suggesting the existence of an order–disorder transition around 770 K associated with the ordering of Cu atoms.

Finally, the effect of the amount of Cu has been analyzed, confirming that a greater amount of Cu naturally intensifies the process associated to ordering of Cu atoms, which, by the way, takes place even in martensite. This is an unprecedented observation, as it has always been assumed that diffusion-related post-quench atomic reordering only takes place in austenite.

## Figures and Tables

**Table 1:** Saturation magnetization of ferromagnetic martensite and austenite obtained by means of the Arrott plots, together with the calculated values for austenite at 0 K.

	10 K (m)		280 K (A)	0 K (A)	
	M_sat_ (emu/g)	M_sat_ (µ_B_)	M_sat_ (emu/g)	M_sat_ ^1^ (emu/g)	M_sat_ ^1^ (µ_B_)
WQS	73.2	2.75	37.4	70.96	2.66
IAS	74.9	2.81	39.6	72.00	2.70
FAS	83.6	3.14	58.3	85.74	3.22

^1^ Calculated values.

## Data Availability

The data presented in this study are available on request from the corresponding author.
